# Exploring supported conversation with familial caregivers of persons with dementia: a pilot study

**DOI:** 10.1186/s40814-019-0398-5

**Published:** 2019-01-18

**Authors:** Joshua Troche, Arielle Willis, Janet Whiteside

**Affiliations:** 1 School of Communication Sciences and Disorders, University of Central Florida, P.O. Box 162215, Orlando, FL 32816 USA; 2 Department of Communication Sciences and Disorders, Baylor University, Waco, TX USA

**Keywords:** Supported conversations, Dementia, Caregivers, Communication

## Abstract

**Background:**

Dementia can lead to difficulties in communication between caregivers and patients. Teaching conversational strategies has been effective for a wide array of clients with acquired neurologic disorders and their caregivers. Research indicates positive results for Supported Conversation for adults with Aphasia (SCA) secondary to stroke. Applying this method to work with caregivers of individuals with dementia could prove to be a valid intervention tool. This investigation examined the applicability of SCA with individuals with dementia and their familial caregivers.

**Method:**

Four dyads (caregiver and individual with dementia) participated in the SCA program with some adaptation for dementia. The program was 4 weeks with a pre-training and post training assessment. The Measure of Skill in Supported Conversation (MSC) and Measure of Level of Participation in Conversation (MPC) were given to measure the overall effectiveness of SCA at teaching and improving communication, respectively. A qualitative analysis of unproductive coping mechanisms also occurred. The Zarit Burden Interview (ZBI) was given to gauge caregiver burden from pre- to post-training.

**Results:**

MSC and MPC scores were significantly improved from baseline to post training, and a significant reduction in unproductive coping behaviors also occured. ZBI scores were variable across participants.

**Conclusions:**

Results suggest that the SCA has the potential to be used to improve communication between persons with dementia and their caregivers. Findings suggest that further research is warranted into the effectiveness of SCA in dementia.

**Trial registration:**

Retrospectively registered 9/5/2018 ISRCTN17622451.

Dementia, although primarily considered a disease that affects working and long-term memory, also has significant effects on language [1]. The disruption in the ability to communicate leads to significant challenges for patients and their professional and familial caregivers [2, 3]. Amongst professional caregivers (i.e., nurses, aids) in institutions, poor communication can often lead to reduced quality of life and reduced psychological and social well-being for patients [4, 5]. In familial caregivers (i.e., spouses, children), poor communication can often lead to difficulties in managing the care of the individual with dementia and earlier placement of the individual with dementia in an institution [6]. For this reason, clinicians often focus on communication as part of the management plan for individuals with dementia. [7]. Interventions in this area range from restorative treatments to maintenance treatments to treatments focusing on training caregivers in communication strategies [8–10]. Overall, however, there remains a paucity of programs that help with the management of communication difficulties in individuals with dementia. This study attempts to adapt a treatment program created for the caregivers of persons with aphasia and for caregivers of individuals with dementia.

## Dementia and communication

Dementia is an umbrella term for numerous chronic disorders in cognitive abilities and declines in memory loss caused by either disease or injury to the brain [11]. In 2007, nearly ten million Americans were caring for someone with dementia, of which the largest proportions were spouses [12]. Dementia can cause a number of language disturbances. Deterioration occurs in semantic memory, phonology, naming, syntax, and discourse [1, 13, 14]. These language-related disturbances often begin mildly but tend to become more severe over time [15–17].

Communication breakdown is regularly listed as one of the top stressors contributing to familial caregiver burden in patients with dementia [18, 19]. In other disorders that cause communication breakdowns, such as stroke and traumatic brain injury, restorative treatment approaches are often the focus of intervention as they work to improve the patient’s communication abilities over time. While restorative treatments have shown some efficacy in dementia populations [9, 20], they are likely not ideal for the degenerative nature of dementia [8]. There has been some success, however, when the burden of treatment shifts from the patient to the caregiver [21, 22].

The majority of individuals with dementia live with a family member in the community [12]. Caregivers often find themselves unprepared for the declines in communication [23, 24]. A variety of studies have looked at the use of communication strategies performed by caregivers and have found that generally there are fewer breakdowns in communication when caregivers employ these strategies versus when they do not [22]. The majority of these studies, however, focus on professional caregivers, as in nurses or paid caregivers, in institutions. Familial caregivers receive much less attention with only a few studies to date examining the use of these communication strategies with familial caregivers in home care settings [18, 21, 25].

## Caregiver training in dementia

Of the programs that have focused on familial caregivers of individuals with dementia, the two programs with the strongest evidence are the FOCUSED program [18, 26, 27] and the TANDEM model [21]. Both programs focus on teaching familial caregivers strategies on how to communicate effectively with an individual with dementia. One of the main focuses of the FOCUSED program is in educating the caregiver on the effects of dementia and also to correct any misconceptions. The program also has an emphasis on methods to improve communication between caregivers and individuals with dementia. These methods are illustrated in the name of the program as FOCUSED is an acronym for these methods: F, functional and face-to-face; O, orient to topic; C, concrete topics; U, unstick any communication blocks; S, structure with yes/no and choice questions; E, exchange conversation; and D, direct, short, simple sentences.

The TANDEM model focuses on breaking down communication into four steps: presentation, attention, comprehension, and remembering. For each of these steps, caregivers are taught to use strategies to optimize communication for caregivers and individuals with dementia. In both programs, caregivers often are trained in small groups with other caregivers.

Both programs have shown efficacy for improving communication between caregivers and individuals with dementia [21, 26]. These communication strategy programs also have a long history in stroke aphasia as well [28]. Kagan, however, argued that while these programs were effective for improving communication, they did not, necessarily, create a feeling of having a natural conversation between the caregiver and individual with aphasia [29]. While the goal of many of the programs was to shift some of the burden of communication to the caregiver, Kagan felt that these programs went too far and could make the person with aphasia become a passive member of the communication partnership [29].

## Supported Conversation for Adults with Aphasia

Supported Conversation for Adults with Aphasia (SCA) offered a possible solution to these problems [29]. The SCA program provides many of the communication strategies seen in other programs that focus on communication strategies but introduces the idea of acknowledging and revealing competence. The theory behind acknowledging and revealing competence is that individuals with aphasia may be reluctant or hesitant to engage in conversation due to the fact that they feel others will think they are incompetent due to their language difficulties. Acknowledging competence is a strategy where the conversation partner performs a series of behaviors where the person with aphasia is reminded both by verbal and nonverbal cues that they remain competent even though they have communication difficulties. These behaviors allow the caregiver to acknowledge that even though the individual with aphasia has had a trauma that has affected their communication, they remain competent overall.

Revealing competence is a set of strategies used by the conversation partner to help the person with aphasia reveal their competence and can be broken down into the sub-components of getting the message in, getting the message out, and verifying the message. Getting the message in requires the caregiver to modify the way the conversation occurs to ensure that the individual with aphasia understands what is being said. Getting the message out requires caregivers to learn strategies that will aid the individual with aphasia in expressing themselves. Verifying the message allows for the individual with aphasia to be valued and understood as the caregiver takes the extra step to summarize what was said in the conversational exchange.

Kagan found that framing the program in these ways leads to not only improved communication but also improved participation from the person with aphasia due to the empowerment of the program by unmasking and revealing their competence [30]. In other words, the naturalness of the conversation is improved because the strategies focus not just on improving communication but also on improving the participation of the person with aphasia in conversation. This more natural conversation style has been shown to improve psychosocial wellness for both persons with aphasia and their caregivers [31].

SCA training modules break down the program into two parts: acknowledging competence and revealing competence. Revealing competence is further broken down into three sub-components: getting the message in, getting the message out, and verifying the message. SCA training modules include videos, discussion questions, prompts for reflection, and role-play scenarios. Concrete examples of behaviors to perform for each of these modules are given throughout these modules. In other words, SCA is a well-organized and implemented program in the world of aphasia.

## SCA and dementia

The FOCUSED program and TANDEM model, while teaching caregivers strategies to improve communication, do not have as strong a focus on improving the participation of individuals with dementia in conversation and acknowledging their underlying competence as seen in the SCA. In other words, the programs focus on improving the extraction of information from the individual with dementia but not the participation of individuals with dementia in conversation, which as we previously noted, was shown to be crucial to creating a more natural conversational dynamic. It is also important to note that communication breakdown between a familial caregiver and individuals with dementia has been shown to be particularly difficult for caregivers leading to psychosocial difficulties and feelings of increased burden [6]. The loss of a longtime conversation partner leads to real detriments to these familial caregivers. Therefore, a program which focuses on both improving communication and participation for individuals with dementia may lead to improved communication but also increased participation for individuals with dementia and decreased burden for their caregivers.

Therefore, in this pilot study, we attempted to adapt the SCA for populations with dementia. After adapting the SCA, we explored the following questions.

1. Can caregivers of individuals with dementia be adequately trained in SCA?
2. Does training lead to improvements in communication and participation between the caregiver and the individual with dementia?
3. Does SCA training lead to reductions in unproductive communication behaviors by caregivers that often lead to frustration and disengagement amongst individuals with dementia?
4. Would SCA training lead to reductions in caregiver burden due to improvements in communication?
5. What are the impressions of the caregivers of this program?

## Method

### Participants

The study was approved, and all participants gave informed consent in accordance with the Institutional Review Board at the University of Central Florida. The participant pool came from Brain Fitness, a strengths-based program that supports individuals experiencing memory loss. The group allows for individuals with mild to moderate dementia to partake in a program that focuses on maintaining skills rather than attempting to rehabilitate lost skills. This site is affiliated with the University of Central Florida’s Communication Disorders Clinic. The participant pool consisted of four dyads, each including a spousal caregiver and their partner with dementia. Dyads were recruited and chosen based on self-reported difficulties in communication between the individual with dementia and their caregiver. Difficulty in communication was defined as an increased difficulty in both exchanging and receiving information between the caregiver and the individual with dementia. All individuals with dementia had been given the Montreal Cognitive Assessment (MOCA) in the last 6 months and had scored between 11 and 21 which is defined as mild to moderate dementia [32]. Participants ranged in age from 69 to 78 (See Table 1 for demographic data for each participant) and had an etiology of Alzheimer’s disease with a primary deficit in memory, not language. All partners with dementia were male, and all spousal caregivers were female.

**Table 1:** Demographic data of individuals with dementia

	Age	MOCA
Dyad 1	78	11
Dyad 2	75	16
Dyad 3	69	17
Dyad 4	72	21

*MOCA* Montreal Cognitive Assessment

### Data collection measures

#### Quantitative measures

The pre-training assessment and post-training assessment conversations were scored using the Measure of Skill in Supported Conversation (MSC) and Measure of Participation in Conversation (MPC) [31]. Ten research assistants were trained by the speech-language pathologist certified in SCA on the SCA program itself and how to score the MSC and MPC. These research assistants were trained over a 2-day period. The raters were blinded as to which videos were taken before the training and which were taken after. The order in which the videos were viewed was also randomized. Each pre- and post-video was rated by all ten research assistants. In other words, each video had ten MPC scores and ten MSC scores. The most common score across the ten participants was considered the consensus score and was the score used for the analysis. Inter-rater reliability ratings of these scores are reported in the “Results” section.

The MSC was used to assess the ability of the caregiver to engage in the principles of supported conversation (research question no. 1). In other words, the measure allowed us to determine how skilled the caregiver became from pre-assessment to post-assessment on the principles of the training program: acknowledging competence and revealing competence. Acknowledging competence can be described as the ability of the individual to acknowledge the competence of an individual with dementia using natural conversation. Some examples of behaviors that acknowledge competence are not patronizing the individual with dementia, maintaining a natural flow/feel to the conversation, and correcting unclear or incorrect responses respectfully. They also include statements like, “I know you’re smart” or “I know you know what you want to say.” Revealing competence requires three things, ensuring the individual with dementia understands the conversation (e.g., short, simple sentences), allowing the individual with dementia to respond or express opinions (e.g., fixed choice or yes/no questions), and verifying the contents of the conversation with the individual with dementia (e.g., reflecting and expanding or “Let me see if I got this right…”). The ability of the individual to acknowledge and reveal competence is scored on a 9-point scale presented as a range of 0–4 with 0.5 levels representing performance level. The score for acknowledging competence and revealing competence is summed together to get the total MSC score.

The MPC was used to assess the level of participation of the individual with dementia in the conversation (research question no. 2). This was done by evaluating the level of interaction and transaction. Interaction can be described as the social connection that is created through the process of discourse. An example would be how well an individual can keep the conversation partner engaged in the conversation or how natural the interaction is between the conversation partners. Transaction is the process of sharing information with your conversation partner. In other words, how well you can take the information or idea that you have in your mind and share that with your partner. Interaction and transaction are both scored on a 9-point scale presented as a range of 0–4 with 0.5 levels representing performance level. These two scores are summed to create the total MPC score.

Both the MSC and MPC have been proven to be valid and reliable in individuals with communication disorders [30, 31, 33]. Various studies have found the reliability of both the MSC and MPC to be greater than .80 [30, 31], and the validity of both measures was supported by work that found that both the MSC and MPC could successfully differentiate between experimental and control groups in experimental studies of the SCA [30, 31].

#### Qualitative measures

In addition to MSC and MPC, a qualitative analysis of the conversations occurred as well. We tabulated the number of instances of joking, quizzing, and volume elevation which is often seen as unproductive coping mechanisms performed by caregivers during conversations and can often cause frustration for individuals with dementia during communication (research question no. 3). Joking was defined as moments in the conversation when the caregiver would make a joke or sarcastic comment that was not meant to include the individual with dementia and was either directed to the clinician, about the individual with dementia, or about the situation. Quizzing was defined as asking multiple open-ended questions in a row without giving the individual with dementia an opportunity to respond. Volume elevation was defined as when the caregiver increased the loudness of their voice to an unnatural volume even though the individual with dementia did not indicate they were having difficulty hearing what was said. Qualitative analysis of the conversations was done by the ten research assistants who scored the dyad conversations using the MSC and MPC. The same analysis procedure was used for the quantitative and qualitative measures: raters were blinded to which videos were taken before the training and which were taken after and the videos were viewed in a randomized order. Each pre- and post-video was rated by all ten research assistants. The most common score across the ten participants was considered the consensus score and was the score used for the analysis.

#### Caregiver measures

The shortened version of the Zarit Burden Interview (ZBI) [34] was used to gauge perceived caregiver burden at the onset and conclusion of the training program (research question no. 4). The shortened version is 12 questions long and is highly correlated with the long version (*r* = .95; 34) which has strong validity and reliability [35, 36]. The short version of the ZBI is given as a questionnaire filled out independently by the caregiver and has questions such as “Do you feel angry when you are around your relative?” or “Do you feel you should be doing more for your relative?” These questions are scored on a scale from 0 to 4 with 0 being never and 4 being nearly always.

#### Social validity

We also included a short survey that collected data about the social validity of the SCA in populations with dementia. Social validity is a measure of the satisfaction that an individual or caregiver has with an intervention. The survey contained three questions; the first question was a 5-point Likert scale question about the benefit of SCA in communication with the individual with dementia. The final two questions were open-ended questions that asked what strategies were most useful and what caregivers thought could be done to improve the program.

### Procedure

The entirety of the experiment took place over a 6-week period. The first week and the sixth week were the pre- and post-assessments which occurred individually with each of the dyads. After the pre-training assessments, the caregivers as a group began a comprehensive training program utilizing the adapted supported conversation for adults training for weeks 2 through 5 for 1 h a week. The training program involved didactic and experiential training methods. Experiential training was, in this context, caregivers going home and practicing the methods learned in the training session with their loved one and then discussing their experience when they returned for the next training session. A licensed speech-language pathologist, trained in SCA, conducted all didactic sessions and led experiential sessions.

#### Pre-training assessment

The first assessment session consisted of each dyad being recorded for baseline data. To achieve this, a 10- to 15-minute conversation was conducted. Topics were chosen to provide a consistent amount of intentional conversation for each dyad for MPC/MSC scoring. Every dyad was asked to answer the following prompt: “Describe the first time you met.” After this prompt, the caregivers could then decide which of the following follow-up prompts to answer: “Do you remember your first home together?” or “Do you have any vacation plans coming up?” The question was posed as a yes/no question, but the caregivers were encouraged to try and get their partners to expand on the yes/no answer. The following prompts allowed us to observe both the interactional and transactional nature of their conversations. The conversations between the caregiver and the individual with dementia were videotaped and audiotaped.

#### Didactic training

**Fig. 1:**
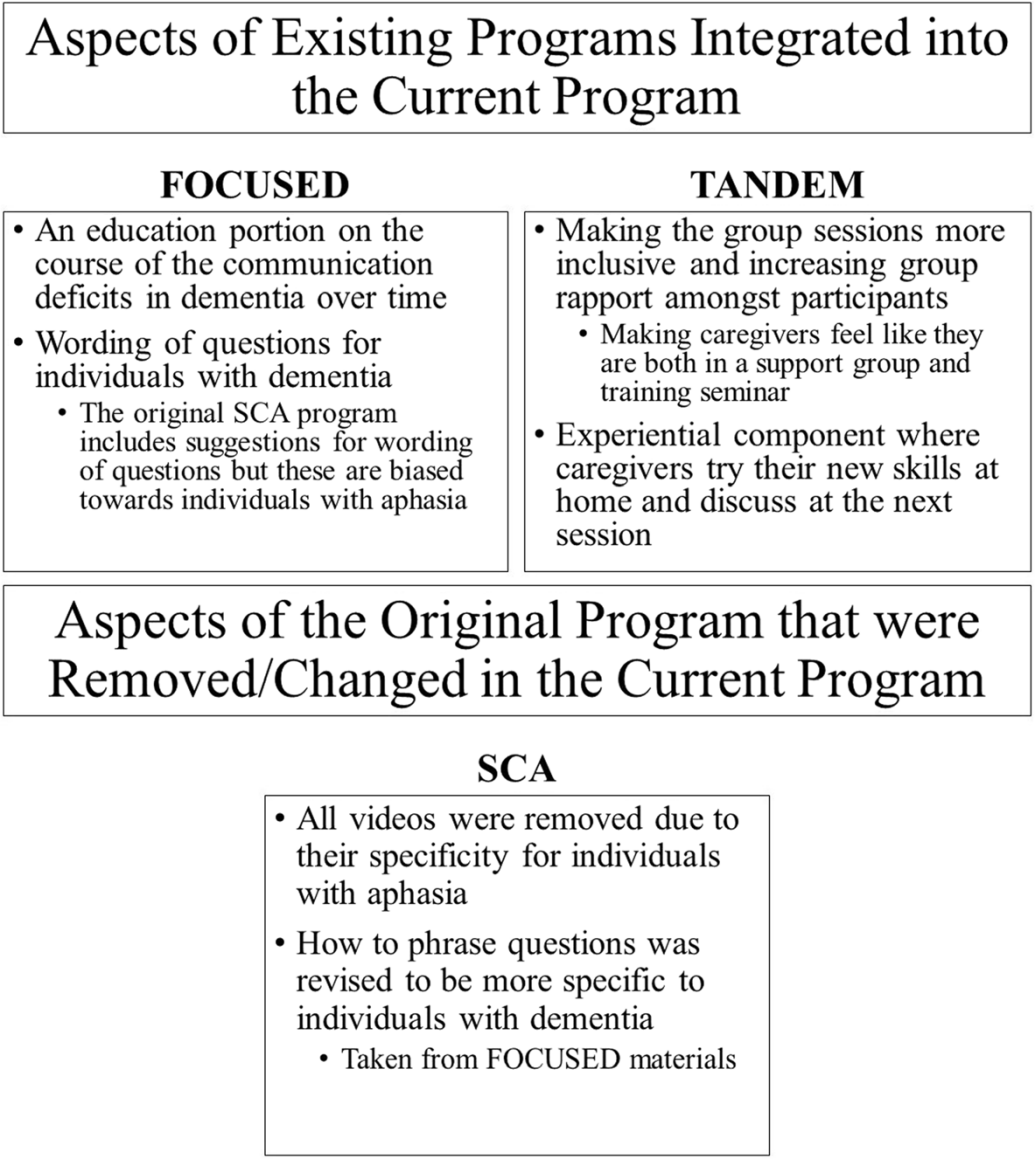
Aspects of the FOCUSED and TANDEM programs that were appended to the SCA program for this study as well as items removed from the SCA to make it more appropriate for individuals with dementia

Materials used in this study were taken and adapted from the learning modules provided in Supported Conversation for Adults with Aphasia. Information from the FOCUSED program and TANDEM model were used to modify portions of the SCA for caregivers of individuals with dementia (see Fig. 1).

Training sessions were broken up into two components similarly to the original materials used in SCA: acknowledging competence [1] and revealing competence [2]. Revealing competence was further broken down into three sub-components: getting the message in, getting the message out, and verifying the message. All information was presented as a slide presentation to the caregivers as a group. All caregivers were present for all four training sessions. Their partners with dementia were not present during the didactic training sessions. What was taught during each session can be found below, for a fuller description see Table 2.

**Table 2:** Description of adapted SCA program

Topic	Description	Skills taught
Introduction, dementia education, and acknowledging competence	1. Trainer and caregivers introduce themselves 2. The SCA program is introduced 3. Typical progression of dementia and its effects on communication 4. How to acknowledge the competence of individuals with dementia	•Group members establish a connection and rapport with other members and the trainer. •Caregivers are educated on how dementia will likely lead to greater difficulties in communication over time. Eliminating distractions and carefully wording questions will become more important as the disease progresses. •Caregivers are taught the importance of acknowledging confidence in improving participation of the individual with dementia in conversation. Acknowledging competence can be achieved by speaking in a natural voice and tone, reassuring their partner in their abilities, and avoiding quizzing. •Caregivers are also taught to take the lead and initiate conversation topics and to throughout interject with expressions of acknowledging competence: I know you’re competent/smart.
Revealing competence and getting the message in	1. Discussion of how home practice of skills went 2. Review of previous topics 3. Description of revealing competence 4. How to get the message in with individuals with dementia	•Caregivers are taught that revealing competence requires getting the information in, getting the correct information out, and verifying the message. •To get the message in, use yes/no questions as much as possible when a longer conversation is not required for communication. Otherwise, use short, simple, and direct sentences without the use of pronouns such as she/him. •Use an expressive voice and easily understood gestures •Write key words on a sheet of paper in large, bold print •Eliminate distractions as much as possible
Getting the message out	1. Discussion of how home practice of skills went 2. Review of previous topics 3. How to get the message out of individuals with dementia	•Caregivers are taught that to help get the message out, caregivers should only ask one question at a time and give the partner adequate time to process and respond. •Focus on the here and now and provide as much context as possible •Ask partner for clues through the use of gesture •Provide paper and pencil
Getting verification of the message	1. Discussion of how home practice of skills went 2. Review of previous topics 3. How to verify the message	•Caregivers are taught that to verify the message they must reflect, expand, and summarize what was said. •Reflecting requires the caregiver to repeat the message. •Expanding requires the caregiver to tell their partner what they think the message was. •Summarizing requires the caregiver to recall and summarize the conversation as a whole.

The first training session began with the distribution of the Zarit Burden Interview (ZBI). Upon collection of the ZBIs, the first training session formally began. During this session, the first learning module was introduced. This module focused on the first component of SCA—acknowledging competence in the person with dementia. This module presented why acknowledging competence is the first step towards better communication between the caregiver and their spouse. It also gave the caregivers examples of how to acknowledge competence such as speaking in a natural voice or tone or reassuring their spouse when they are struggling. The videos that are usually presented in the SCA program were removed due to their specificity for individuals with aphasia. In the FOCUSED program, there is a considerable focus on education of dementia and its effects. During this first session, we appended the program by adding information about dementia and the common communication breakdowns seen in the disorder. We also included information about common complaints from individuals with dementia about communication with their spouses such as frequent quizzing and dominating conversations and how these behaviors can be adjusted to aid in acknowledging competence. After this initial training session, each subsequent session was lead with a review of the module from the week before reinforcing key concepts. This was taken from the work on the TANDEM model that found that review of concepts from previous sessions helped carryover of information to new sessions.

Module two was presented during the second week of the training program and introduced the second component of SCA—revealing the inherent competence of persons with dementia. This session primarily focused on creating opportunities to get the message across to the person with dementia. This included writing keywords in bold print and using short, simple sentences. In addition to the methods provided in the SCA materials, information about sharing the floor, eliminating distractions, and framing conversations to highlight competence were added.

During week 3 of the training program, another sub-component was introduced—finding ways to help individuals with dementia get the message out such as providing a pen and paper to write keywords. Adjustments made to the original material in this section included providing a context and focusing on the present when communicating with a person with dementia. Using a hierarchy of questions was added and expanded to provide a loose guideline in interactions with persons with dementia. The idea of a hierarchy of questions comes from the work of the FOCUSED program.

In the final week of the training program, the caregivers were presented with the last SCA sub-component in revealing competence—getting verification from persons with memory impairment. No adjustments to the material were necessary for this learning module. Emphasis was placed on three main ideas in this module: reflecting, expanding, and summarizing. The caregivers were taught to repeat the message to their partner, explain what the perceived idea of the message was, and then summarize the conversation with their partners with dementia to ensure that they were understood. The Zarit Burden Interview was distributed and collected for a second time towards the conclusion of the final training session.

#### Experiential training

Concurrently, during the training program, the caregivers were asked to actively engage in using SCA with their spouses at home as “homework.” The caregivers were asked to relay feedback at the beginning of each session to provide a meaningful discussion regarding what was and was not successful. The speech-language pathologist addressed concerns and aided in identifying behaviors that either facilitated or hindered effective communication.

#### Post-training assessment

Upon completion of the program, a second assessment session was conducted for week 6. The same set of transactional and interactional conversation questions was videotaped and audiotaped for post-assessment using the quantitative measurements of the MSC and MPC.

### Statistical analysis

Considering the small sample size in this pilot study, descriptive statistics were deemed most appropriate for analyzing the pre- and post-differences in the MSC/MPC measures, the measure of unproductive behavior, and the ZBI. A correlation was also run to determine if changes in MSC scores led to changes in MPC scores and to determine if changes in MSC or MPC scores were related to reductions in unproductive behaviors or caregiver burden.

## Results

### Reliability measures

The intraclass correlation, a reliability measure, was *r* = 0.94 for the MSC, *r* = 0.92 for the MPC, and *r* = 0.87 for the qualitative measure which, according to [37], is excellent inter-rater reliability.

### Descriptive statistics

Due to limited sample size, descriptive statistics were employed to examine differences between our outcome measure scores from pre-training to post-training There was an average increase of 1.63 (SD = 1.03) on the MSC and 1.88 (SD = .63) on the MPC from pre-training to post-training (Table 3). It was also noted that there was a reduction in unproductive behaviors including joking (M = − .18; SD = .11), quizzing (M = − .22; SD = .15), raising volume (M = − .03; SD = .1), and unproductive behaviors overall (M = − .43; SD = .3; see Table 4) Caregiver burden as measured by the ZBI (M = − .5; SD = .7.57) was reduced overall from pre- to post-training (although note individual differences; Table 3).

**Table 3:** Pre- and post-scores for the MSC, MPC, and ZBI

	MSC			MPC			ZBI		
	Pre	Post	Δ	Pre	Post	Δ	Pre	Post	Δ
Dyad 1	4	7	3	2.5	5	2.5	23	23	0
Dyad 2	3.5	5	1.5	4	6	2	26	26	0
Dyad 3	4.5	6	1.5	4	6	2	25	21	− 4
Dyad 4	6.5	7	0.5	7	8	1	25	9	− 16
Mean	4.6	6.3	1.63	4.4	6.3	1.88	25	20	− 5
SD	1.3	1	1.03	1.9	1.3	0.63	1.3	7.5	7.57

*MSC* Measure of Skill in Supported Conversation, *MPC* Measure of Participation in Conversation, *ZBI* Zarit Burden Interview, *Δ* change from pre- to post-traning

**Table 4:** Pre- and post-scores for the Qualitative Measure

	Joking			Quizzing			Raising Volume			Total		
	Pre	Post	Δ	Pre	Post	Δ	Pre	Post	Δ	Pre	Post	Δ
Dyad 1	0.38	0.06	− 0.31	0.50	0.06	− 0.44	0.13	0.00	− 0.13	1.00	0.13	− 0.88
Dyad 2	0.33	0.14	− 0.19	0.85	0.75	− 0.10	0.00	0.00	0.00	1.18	0.89	− 0.29
Dyad 3	0.17	0.00	− 0.17	0.33	0.17	− 0.16	0.00	0.00	0.00	0.50	0.17	− 0.33
Dyad 4	0.15	0.11	− 0.04	0.29	0.11	− 0.17	0.00	0.00	0.00	0.44	0.22	− 0.21
Mean	0.26	0.08	− 0.18	0.49	0.27	− 0.22	0.03	0.00	− 0.03	0.78	0.35	− 0.43
SD	0.1	0.1	0.11	0.3	0.3	0.15	0.1	0	0.06	0.4	0.4	0.3

*Δ* change from pre- to post-training

### Individual differences in response SCA treatment

**Fig. 2:**
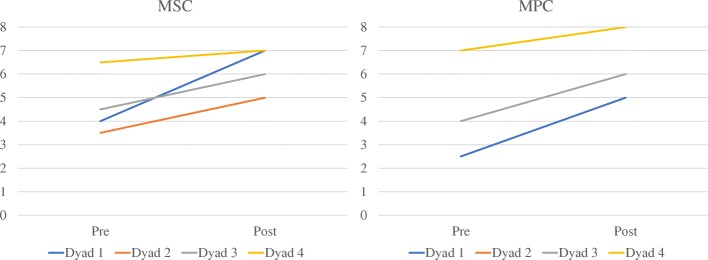
Line graph of the MSC and MPC scores for each dyad from pre- to post-traning. Note that dyad 2 and 3 had the same score on the MPC and therefore overlap. The color for dyad 3 is shown but not dyad 2

We also examined individual differences in response to the SCA by examining individual differences in MSC and MPC scores pre- and post-training across participants (see Fig. 2). It should be noted all caregivers displayed increases in the MSC and all individuals with dementia showed improvements in MPC scores from pre- to post-training. The slope of the change, however, was different for each participant. Overall, the dyads who started lower on each scale tended to have the steepest slopes, while the dyad who started at the very top (dyad 4) had the shallowest slope for both measures.

All participants saw reductions in unproductive behaviors from pre- to post-training overall and in the individual behaviors as well. The only behavior which did not see a reduction across all dyads was raising volume. This is unsurprising however as dyad 2, 3, and 4 had no instances of this behavior at the pre-treatment assessment. ZBI scores had high levels of individual difference with some dyads having no change in burden (dyad 1 and 2), one having a small decrease in burden (dyad 3), and one having a large decrease in burden (dyad 4). No dyads saw an increase in ZBI scores.

### Correlation between outcome measures

Changes in score from pre- to post-training in the MSC and MPC measures were highly positively correlated (*r*_τ_ = 1; Table 5). The greater the change in MSC from pre- to post-training, the greater the improvement in MPC. Change from pre- to post-training in MSC and MPC was also highly negatively correlated with reductions in unproductive behaviors (*r*_τ_ = − .91). The larger the improvement in MSC and MPC, the greater the reduction in unproductive behaviors.

**Table 5:** Correlation table of outcome measures

	MSC	MPC	ZBI	UnBeh
MSC	1	1	0.80	− 0.91
MPC	1	1	0.80	− 0.91
ZBI	0.80	0.80	1	0.55
UnBeh	− 0.91	− 0.91	− 0.55	1

*MSC* Measure of Skill in Supported Conversation, *MPC* Measure of Participation in Conversation, *ZBI* Zarit Burden Interview, *UnBeh* unproductive coping behaviors

### Social validity

Overall, the caregivers felt the SCA was a very beneficial program (Table 6). Regarding what strategies were most helpful, the caregivers most often mentioned sharing the floor, asking yes/no questions, writing keyword/phrases, and giving time to respond. The caregivers also suggested that the program could be improved if it were longer. They felt rushed at times with the information and felt a longer session or more weeks for the program would have been beneficial. They also suggested videos of the SCA in action with individuals with dementia. These videos do exist for the SCA, but are with individuals with aphasia, not individuals with dementia.

**Table 6:** Caregivers self-reported benefit from SCA program

	Benefit
Dyad 1	5
Dyad 2	4
Dyad 3	5
Dyad 4	5

Benefit: Was the program beneficial to your communication with your spouse? “1 = Definitely not” to “5 = definitely yes”

## Discussion

This pilot study is an initial attempt at adapting Supported Conversation for Adults with Aphasia to caregivers of individuals with dementia. We attempted to begin to answer the following research questions:

1. Can caregivers of individuals with dementia be adequately trained in SCA?
2. Does training lead to improvements in communication and participation between the caregiver and the individual with dementia?
3. Does SCA training lead to reductions in unproductive communication behaviors by caregivers that often lead to frustration and disengagement amongst individuals with dementia?
4. Would SCA training lead to reductions in caregiver burden due to improvements in communication?
5. What are the impressions of the caregivers of this program?

Our pilot study suggested that caregivers can successfully learn the SCA as evidenced by improvements in MSC scores and then apply the training to improve communication and participation between themselves and the individual with dementia which was indicated by improvements in MPC scores. The SCA has been used in aphasia [29, 30] and adapted for TBI [33], but this is the first instance of an adaptation that focuses on individuals with dementia, and these initial findings suggest that the program may be effective in improving communication and participation between individuals with dementia and their caregivers. While the TANDEM program has been shown to enhance the quality of life of caregivers and not increase caregiver burden [21], it was never determined if the program could improve the exchange of information between the caregiver and individual with dementia as was shown in this study. Exchange of information was analyzed for the FOCUSED program [26], but it was not determined whether the individual with dementia was actually more engaged and participating in the conversation. This study analyzed this question and found that the SCA did improve engagement and participation between caregivers and individuals with dementia.

Individuals with dementia note unproductive communication behaviors as one of the more frustrating aspects of communicating with caregivers [38]. Studies into the FOCUSED and TANDEM model have not focused on these behaviors. Our qualitative analysis of the sessions revealed that unproductive communicative behaviors were reduced by the program and were associated with improvements in communication and participation between caregivers and their partners with dementia. Overall, caregivers felt the program was beneficial in improving the communication between them and their partner.

The effect of the SCA on caregiver burden remains unlcear. Caregiver burden is a multifactorial phenomenon [6]. While difficulties in communication have been shown to lead to increases in caregiver burden, these difficulties are often not the only source of burden. Some participants saw large reductions in caregiver burden, while others saw small or no reductions in caregiver burden. These findings are similar to those found in work on the TANDEM intervention [21]. It should be noted, however, that no dyads saw an increase in caregiver burden.

The SCA program centers on teaching the caregiver how to acknowledge the competence of the individual with dementia and reveal their competence. This is what makes the SCA different than other programs such as FOCUSED or TANDEM. While all three teach the caregivers communication strategies to improve communication, the SCA allows for greater participation. As Kagan [29] describes, the SCA allows for caregivers and individuals with dementia to feel as though they are having an adult conversation. Our findings suggest that the more caregivers use the techniques of SCA, the greater the improvement in communication and participation. Increased use of SCA techniques also led to reductions in unproductive communication behaviors which would often lead to frustration in the individuals with dementia.

### Limitations of the study

This was a pilot study, and therefore, there were significant limitations in this work. The sample size was small, and the sample only contained males with dementia; therefore, there is some selection bias with this sample. We also focused on individuals with mild to moderate dementia; therefore, it is unclear how this work might be extrapolated to more severe diagnoses. Initial data suggests the moderate diagnoses with the greater communication difficulties saw the steepest slope change, but our power remains too low to extrapolate across the severity spectrum. Also, individuals who participated in this study continued to participate in activities at the Brain Fitness program leading to the possibility of some cointervention effects.

### Future research

The findings of this work suggest that future work with larger samples is warranted. Future research into the questions of SCA in dementia must address the limitations described above. There should be an increase in the sample size with a more diverse set of participants and caregiver situations (in this study, all dyads were husband and wife). It would also be of interest to investigate how we must adapt the SCA for the continuing progression of the disease. Are there techniques that are more appropriate for a milder case as compared to a more severe case? As stated above we have some insight into this question for mild and moderate levels but none for more severe levels of dementia. In that same vein, it will be important to continue to investigate how to adapt the SCA so it leads to the best results for individuals with dementia.

Dosage is another important future research question. Caregivers noted that they wanted more time with the program; it will be important to determine what length of treatment will lead to maximal benefits while also making sure not to increase caregiver burden. Also, the creation of videos of the SCA in use in populations with dementia would also likely be of benefit to the program.

It would also be of interest to analyze discourse in a more robust manner in future studies. A measure such as Correct Information Unit (CIU), which has been used in other studies on communication between caregivers and individuals with dementia [39], could be a strong measure of the quality of the discourse amongst these individuals with dementia and their caregivers.

In review, this study represents an initial attempt to adapt Supported Conversation for Adults with Aphasia in caregivers for adults with dementia. Furthermore, this investigation provides the groundwork for future studies and provides a platform for discussion in regards to bringing about positive change to interactional communication strategies utilized in individuals with dementia. The results suggest an SCA training program for caregivers of individuals with dementia can lead to improvements in communication and participation. This study serves as a basis for future exploration in Supported Conversation for Adults with Aphasia with caregivers of individuals with dementia.

## Abbreviations

MOCA: Montreal Cognitive Assessment
MPC: Measure of Participation in Communication
MSC: Measure of Skill in Supported Conversation
SCA: Supported Conversation for Adults with Aphasia
ZBI: Zarit Burden Interview
